# Comparison of Molecular Subtypes of Carcinoma of the Breast in Two Different Age Groups: A Single Institution Experience

**DOI:** 10.7759/cureus.2834

**Published:** 2018-06-18

**Authors:** Pooja Gupta, Naresh N Rai, Lakshmi Agarwal, Swati Namdev

**Affiliations:** 1 Pathology, National Institute of Pathology, New Delhi, IND; 2 Pathology, Government Medical College, Kota, IND

**Keywords:** immunohistochemistry, molecular subtyping, prognosis, luminal a, luminal b, triple negative, er, pr, breast carcinoma, her2

## Abstract

Background

Hormonal analysis and molecular subtyping are used as an important predictive and prognostic factors in women with carcinoma of the breast. The aim of this study was to analyze and compare the hormonal (estrogen receptor (ER) and progesterone receptor (PR)) and human epidermal growth factor (HER2) status among women with carcinoma breast belonging to two different age groups and classify them in molecular subtypes (luminal A, luminal B, triple negative, and HER2).

Materials and Methods

This was an analytical cross-sectional study performed at a tertiary care center in Northern India. Breast carcinoma cases treated over a period of two years were stratified into two groups (≤ 40 years: younger group, n = 27 and > 40 years: older group, n = 33). Their hormonal (ER, PR) and HER2 status were studied using immunohistochemistry (IHC) and classified according to the molecular classification of the breast carcinoma.

Results

A total of 60 cases of breast carcinoma were treated for hormonal and HER2 status during our study period and were classified into four subtypes. In the younger group (n = 27), luminal A (n = 16, 59.2%) was the most common molecular subtype, followed by triple negative (n = 6, 22.2%), HER2 (n = 4, 14.8%), and luminal B (n = 1, 3.7%). Similarly, in the older group luminal A (n = 20, 60.6%) ranked first, followed by triple negative (n = 10, 30.3%), HER2 (n = 2, 6.0%), and luminal B (n = 1, 3.0%).

Conclusion

Carcinoma of the breast in young women shows variation in the prevalence of molecular subtypes in different regions of the world. The results of our study are in accordance with the Asian literature, showing no significant difference in molecular subtyping of carcinoma breast in younger versus older women. More molecular research is needed to clearly understand the pathophysiology associated with carcinoma of the breast in young women.

## Introduction

Globally, carcinoma breast accounts for the most common type of malignancy in women. It is an extremely heterogeneous disease, resulting from the interaction of both inherited and environmental risk factors. Age, marital status, menstrual history, diet and lifestyle factors, hormonal exposure, and family history are important factors in the etiopathogenesis of breast carcinoma. Prognosis depends on multiple clinical, pathological, and molecular factors. These include histological type, histological grade, lymphovascular invasion, lymph node metastases, and the status of hormonal receptors—estrogen receptor (ER), progesterone receptor (PR), and human epidermal growth factor (HER2) status of the tumor. Current treatment strategies rely on the characterization of the hormone receptors ER/PR protein expression status and the HER2 protein expression or gene amplification [1-2]. Breast carcinoma tends to be more advanced, with inferior survival and higher recurrence rates in young women compared to older women. Risk factors, clinical outcomes, and tumor biology of the breast carcinoma are different in these women (≤ 40 years of age), suggesting that it represents a distinct entity altogether [3].

To the best of our knowledge, a study showing the variation in the prevalence of molecular subtypes in young and old subjects with breast carcinoma has not been reported from India. This study aims to characterise the differences in hormonal and molecular subtyping of carcinoma of the breast in the two different age groups.

## Materials and methods

This analytical cross-sectional study was conducted from January 2015 to December 2016 at a tertiary care center in Northern India after obtaining approval from the Institute Ethics Committee. The study population comprised of all diagnosed carcinoma of the breast cases, received for hormonal and HER2 status. The cases were divided into two groups on the basis of their age:  ≤ 40 years (younger group) and > 40 years (older group). Histological type, grade, lymph node involvement, and lymphovascular invasion were separately evaluated for correlation.

Immunohistochemistry

Representative formalin-fixed, paraffin-embedded sections of tumor and the adjacent normal breast tissue (internal control) were processed for ER, PR, and HER2 immunohistochemistry (IHC) staining. Antigen retrieval was done with Tris-EDTA buffer (pH = 9) and the slides stained with monoclonal antibodies against estrogen and progesterone receptors by a labeled streptavidin-biotin (LSAB) system (ER Clone ID5 and PR Clone IA6, DAKO). HER-2 staining was done with a polyclonal antibody against HER2 oncoprotein (DAKO). All the immunostained slides were reviewed and evaluated as per the current American Society of Clinical Oncology (ASCO)/College of American Pathologists (CAP) guidelines [1-2]. Cases were then classified into different molecular subtypes.

Data collection

Continuous variables were described as the mean and standard deviation (SD) while the categorical variables were stated as percentages.

## Results

A total of 60 breast carcinoma cases were received for IHC over a period of two years. Mean (± SD) age was 46.6 (± 3.42) years with a maximum incidence in the fourth decade (41.7%) and fifth decade (26.7%) (Table 1). 

**Table 1:** Age Distribution of Cases in the Study

Age (years)	# of patients (%)
21 - 30	02 (3.3)
31 - 40	25 (41.7)
41 - 50	16 (26.7)
51 - 60	05 (8.3)
61 - 70	12 (20)
Total	60 (100)

The mean (± SD) age in the younger group (n = 27) was 37.3 (± 3.4) years, while it was 54.2 (± 8.3) years in the older group (n = 33). Invasive carcinoma of no special type (NST) was the most common histological subtype (n = 54, 90.0%). Other subtypes documented were lobular carcinoma, colloid carcinoma, tubular carcinoma, medullary carcinoma, and infiltrating ductal carcinoma (IDC) with neuroendocrine differentiation (Table 2).

**Table 2:** Distribution of Histopathological Subtypes of Breast Carcinoma in the Study NST: no special type; IDC: invasive ductal carcinoma

Histological subtype	# (%)
Invasive Carcinoma NST	54 (90.0)
Lobular carcinoma	2 (3.3)
IDC with neuroendocrine differentiation	1 (1.7)
Tubular carcinoma	1 (1.7)
Medullary carcinoma	1 (1.7)
Colloid carcinoma	1 (1.7)

The tumors were classified into four groups (luminal A, luminal B, HER2, and triple negative) by their ER/PR/HER2 profile: (a) ER+ and/or PR+ but HER2-; (b) ER+ and/or PR+ and HER2+; (c) ER- and/or PR- but HER2+; and (d) ER-, PR- and HER2- (triple-negative). Overall, luminal A (n = 36, 60.0%) was the most common molecular subtype, followed by triple negative (n = 16, 26.7%), HER2 (n = 6, 10.0 %), and luminal B (n = 2, 3.3%) (Table 3).

**Table 3:** Distribution of Molecular Subtypes of Breast Carcinoma Among the Two Age Groups HER2: human epidermal growth factor receptor-2

Age group	Luminal A	Luminal B	HER2	Triple negative
Older	20 (60.6%)	1 (3.0%)	2 (6.0%)	10 (30.3%)
Younger	16 (59.2%)	1 (3.7%)	4 (14.8%)	6 (22.2%)
Total	36 (60.0%)	2 (3.3%)	6 (10.0%)	16 (26.7%)

In the younger group, luminal A (n = 16, 59.2%) was the most common molecular subtype followed by triple negative (n = 6, 22.2%), HER2 (n = 4, 14.8%), and luminal B (n = 1, 3.7%). Similarly, in the older group, luminal A (n = 20, 60.6%) ranked first, followed by triple negative (n = 10, 30.3%), HER2 (n = 2, 6.0%), and luminal B (n = 1, 3.0%). Lymph node metastases were documented in 27 (45.0%) cases. Prevalence of nodal metastases was the highest in the HER2 molecular subtype (Table 4).

**Table 4:** Comparison of Metastatic Pathology in Older and Younger Age Groups According to Their Molecular Subtypes HER2: human epidermal growth factor receptor-2

Age group	Luminal A	Luminal B	Her 2	Triple negative	Total
Older	5 (25%)	1 (100%)	2 (100%)	6 (60%)	14 (42.4%)
Younger	8 (50%)	0	2 (50%)	3 (50%)	13 (48.1%)

Lymph node metastases in the younger and older groups were seen in 13 (48.1%) and 14 (42.4%) cases, respectively. Overall, lymphovascular invasion was seen in 10 (16.7%) cases, being more common in the younger group compared to the older group (Table 5).

**Table 5:** Comparison of Metastatic Lymph Nodes and Lymphovascular Invasion in the Two Age Groups

Age group	Metastatic lymph nodes	Lymphovascular invasion
Younger	48.0%	26.0%
Older	42.0%	9.1%
Total	45.0%	16.7%

**Figure 1:**
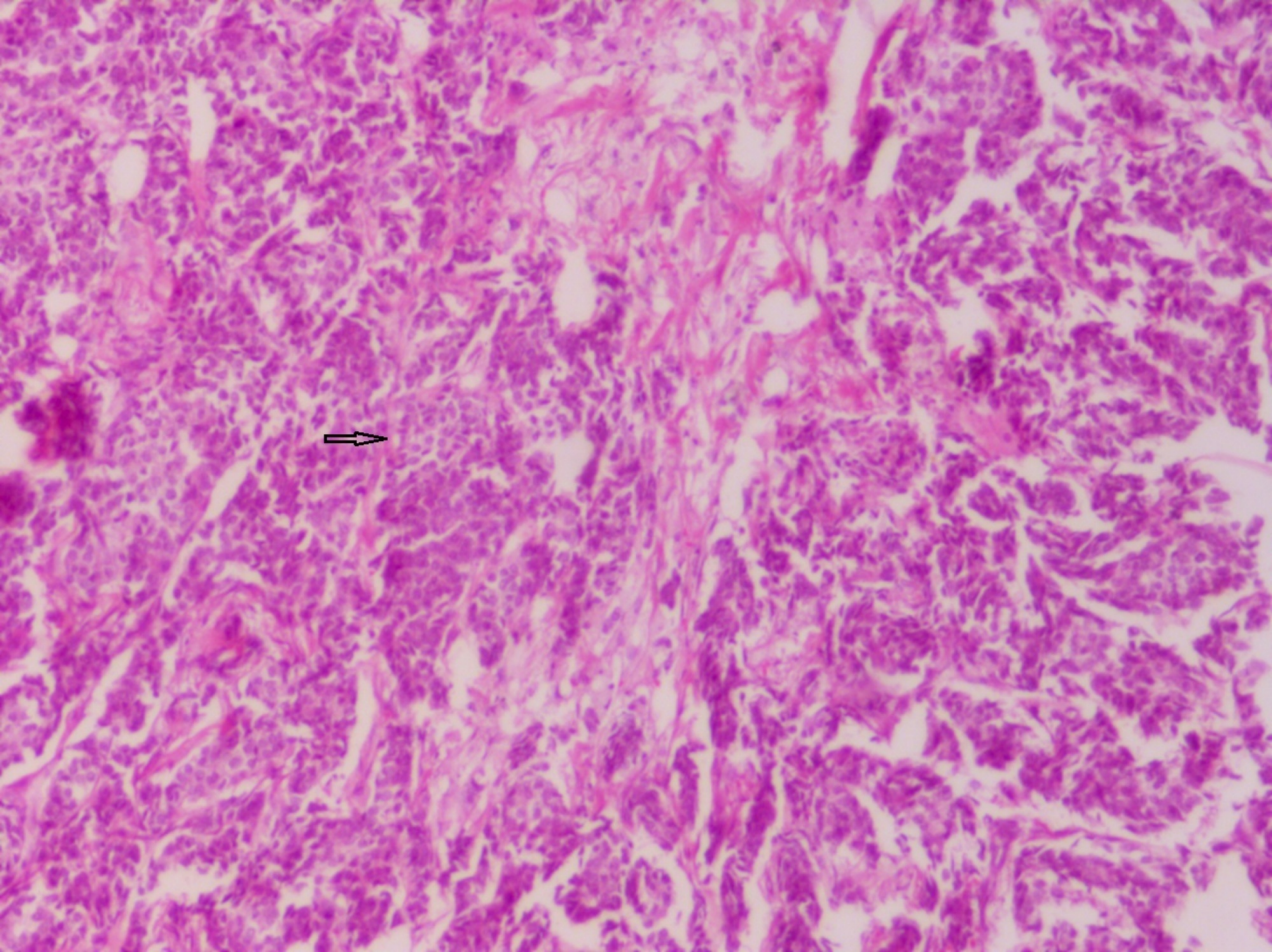
Section showing invasive carcinoma NST Sheets and cords of closely packed carcinoma cells infiltrating into fibrocollagenous stroma (hematoxylin & eosin stain 100x) NST: no special type

**Figure 2:**
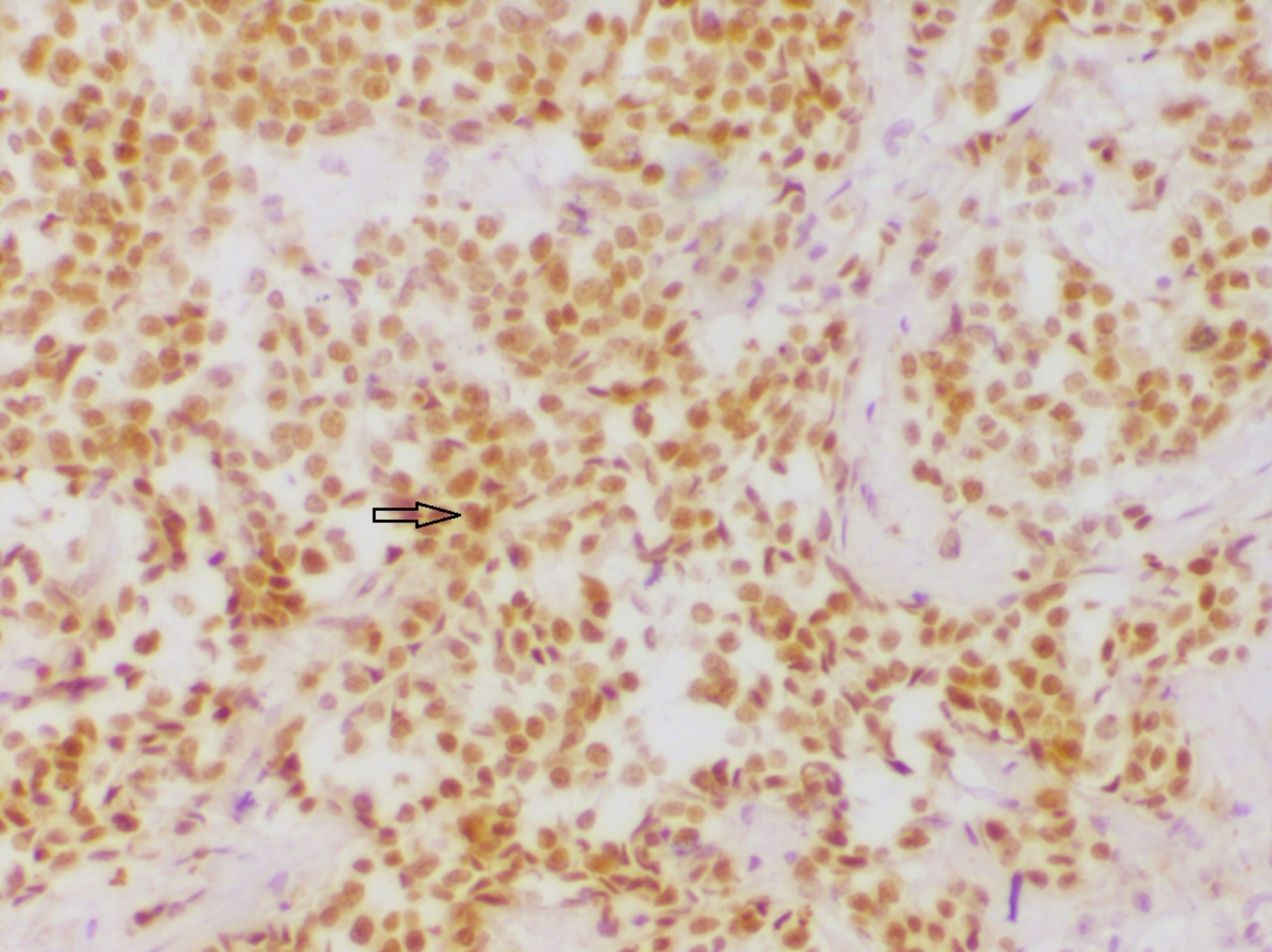
Section showing estrogen receptor (ER) positivity ER immunohistochemistry showing strong nuclear reactivity (200x).

**Figure 3:**
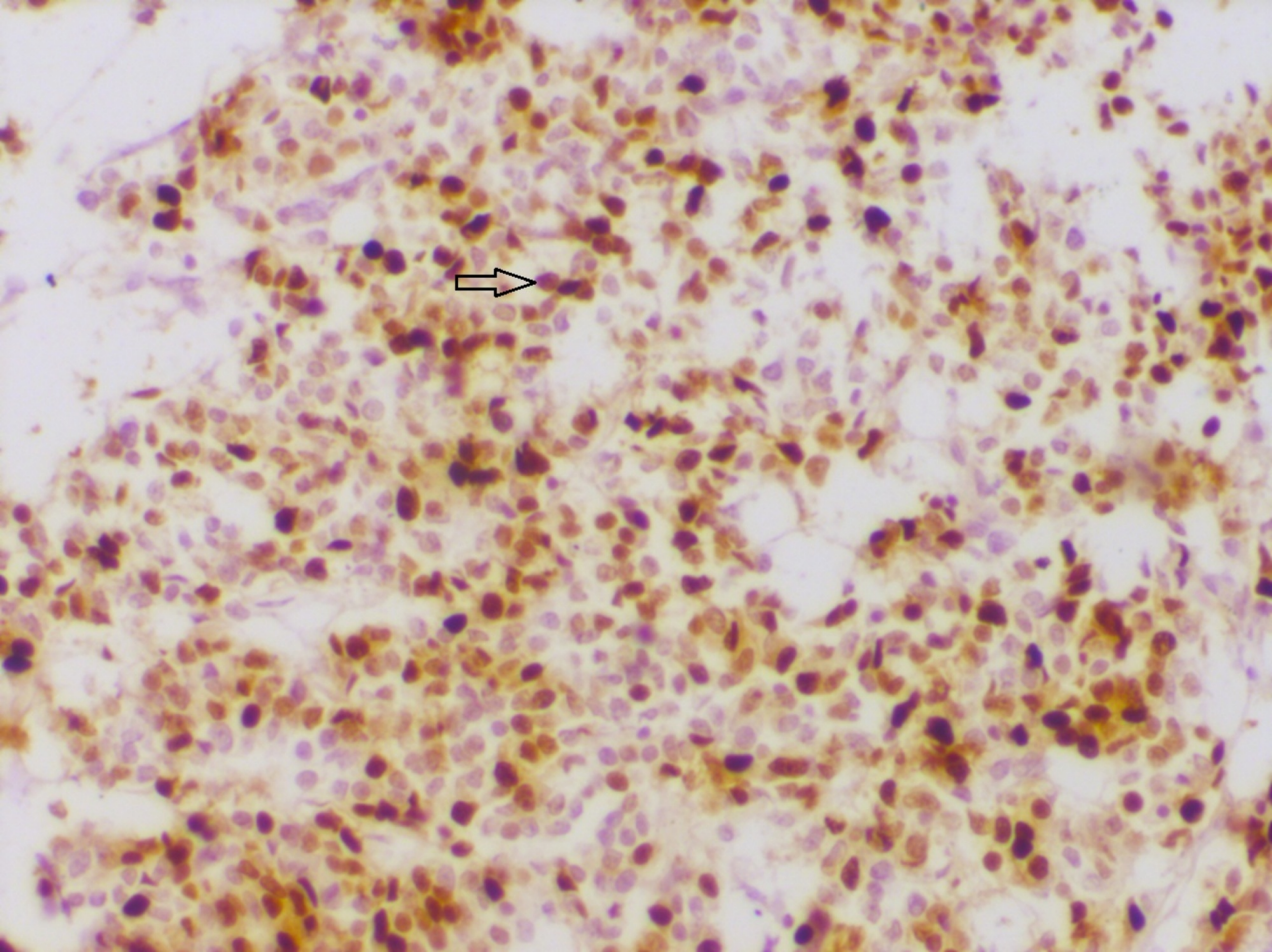
Section showing progesterone receptor (PR) positivity PR immunohistochemistry showing strong nuclear reactivity (200x)

**Figure 4:**
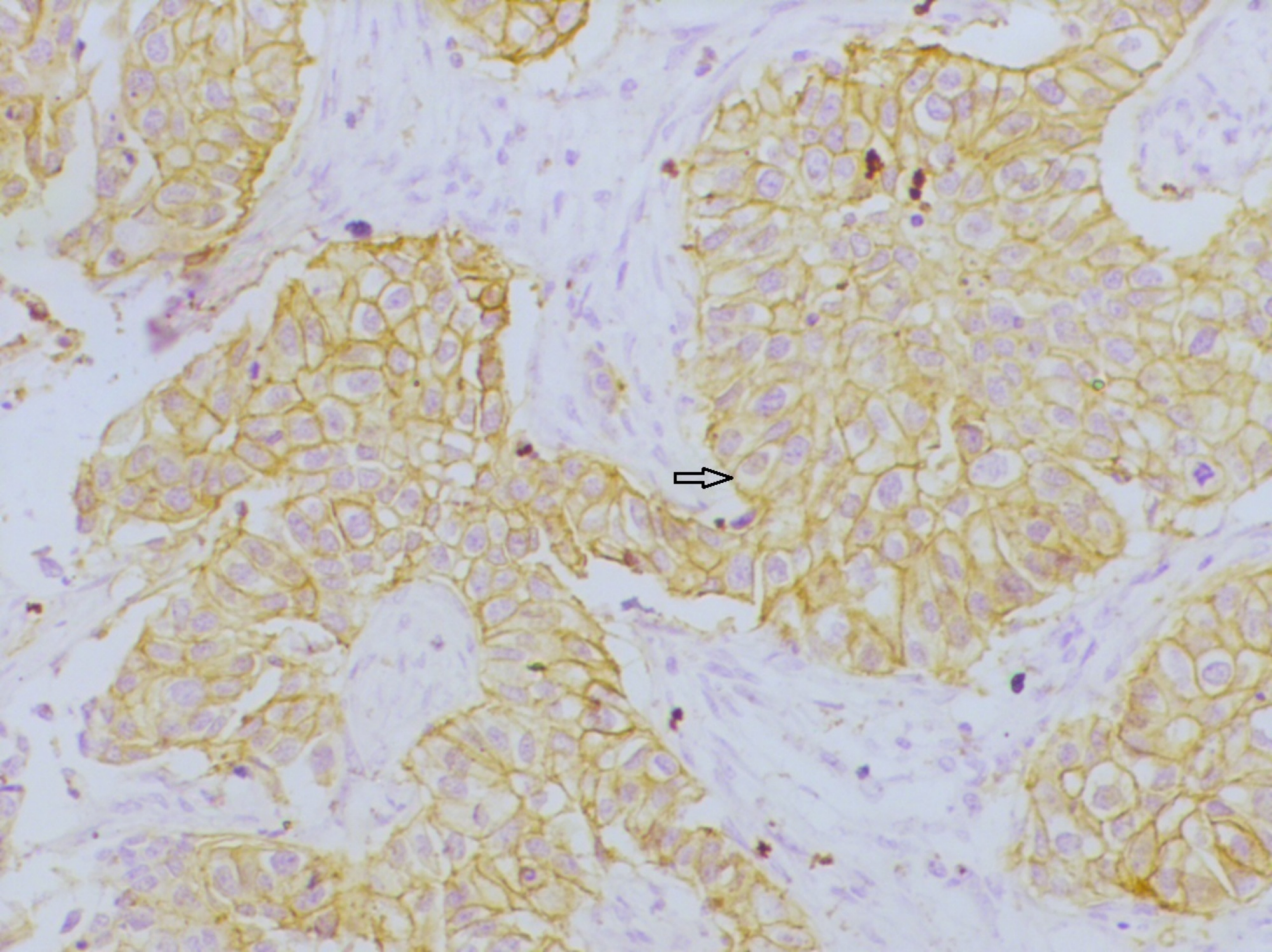
Section showing human epidermal growth factor receptor-2 (HER2) positivity HER2 immunohistochemistry showing intense membrane reactivity (200x)

**Figure 5:**
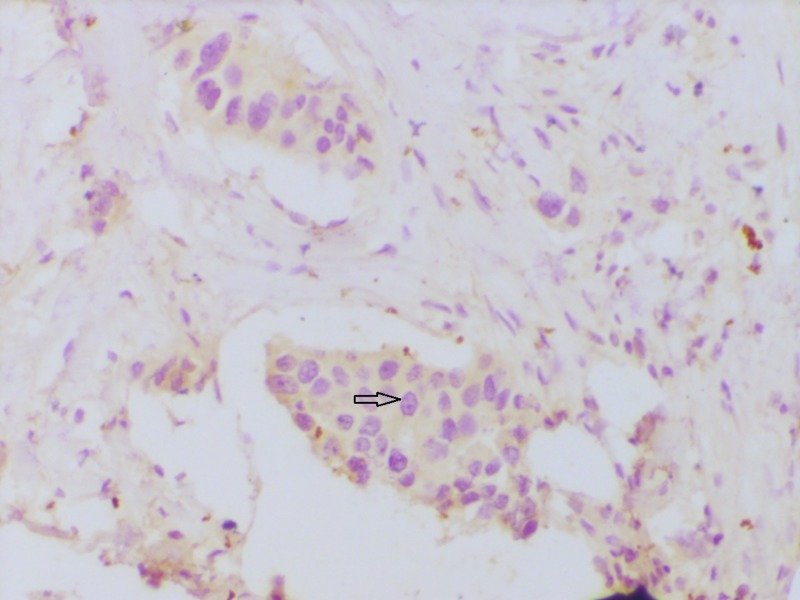
Section showing estrogen receptor (ER) negativity ER immunohistochemistry showing absent nuclear reactivity (200x)

**Figure 6:**
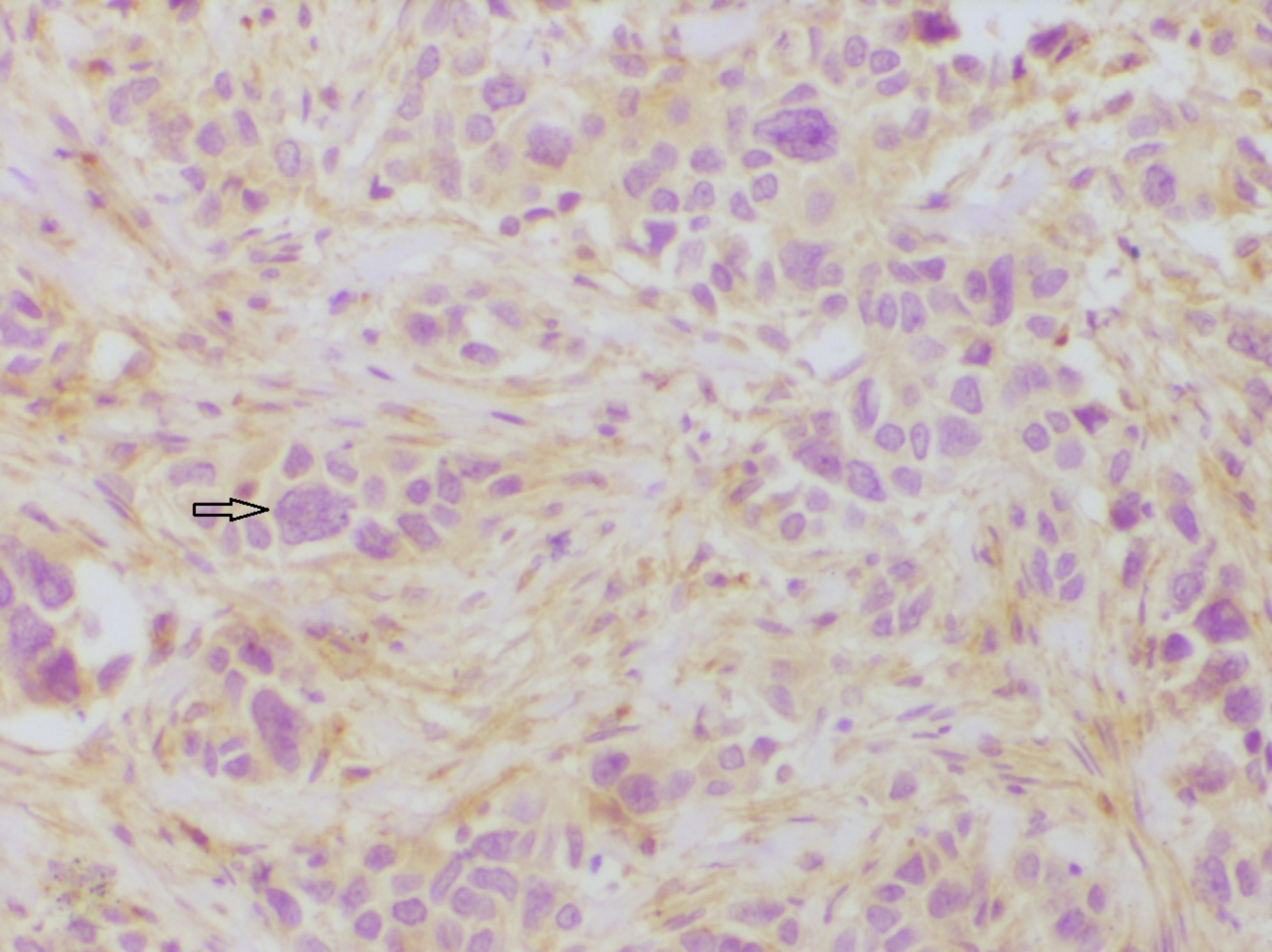
Section showing progesterone receptor (PR) negativity PR immunohistochemistry showing absent nuclear reactivity (200x).

**Figure 7:**
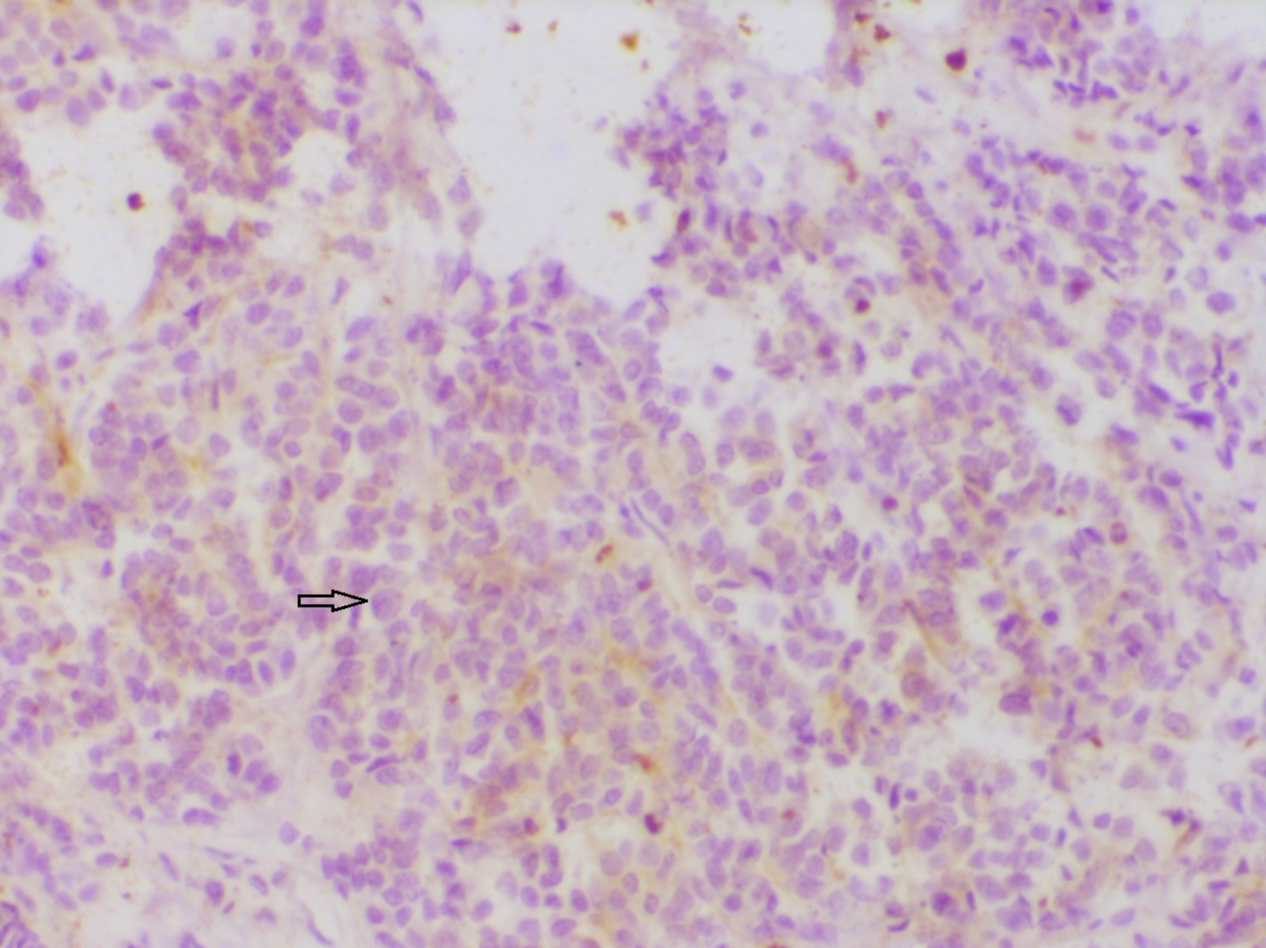
Section showing human epidermal growth factor receptor-2 (HER2) negativity HER 2 immunohistochemistry showing  absent  membrane reactivity (200x)

Some of the representative IHC microphotographs from the study are shown in Figures 1-7.

## Discussion

Breast carcinoma pathophysiology involves the complex interplay of a number of variables. Assessment of its prognosis and predictive outcomes requires the understanding of both clinicopathological and molecular factors. Our study focuses on the unique pathogenesis of carcinoma breast in younger versus older women and tries to elucidate the differences in molecular subtyping between them.

There is no consistent uniformity in molecular subtyping in different regions of the world, suggesting a need to apprehend young breast carcinogenesis in our setting [3]. The mean age in the younger group and older group in our study was 37.3 and 54.2 years, respectively. This was similar to the study done by Alzaman et al. [4], where the mean age in the younger and older groups was 36 and 55 years, respectively. Invasive carcinoma NST was the most common histological subtype, followed by lobular carcinoma, tubular carcinoma, medullary carcinoma, colloid carcinoma, and IDC with neuroendocrine differentiation. This was in accordance with a study done by Makki [5], which reported IDC as the commonest subtype. At a molecular level, luminal A was the most common molecular subtype, followed by triple negative, HER 2, and luminal B, which is comparable with a study done by Alnegheimish et al. [6], where the most prevalent subtype was luminal A (58.5%), followed in descending order of frequency by triple negative (14.8%), luminal B (14.5%), and HER2 (12.3%). The prevalence of molecular subtypes in older women was consistent with the study by Alzaman et al. [4], showing luminal A as the most common subtype (51.6%).

Interestingly, in our study, luminal A was the most common molecular subtype in younger group followed by triple negative, HER2, and luminal B. However, studies of breast carcinoma in younger women belonging to African or American ethnicity show HER2, triple negative, and luminal B subtypes to be more common compared to luminal A, indicating aggressive disease, higher grade, and poorer prognosis [7]. The results of our study are comparable to that done by Lin et al. [8] from Taiwan in concluding that younger patients had a significantly higher prevalence of luminal A and lower prevalence of triple negative-like subtypes. Studies by Kurebayashi et al. [9] from Japan and Kumar et al. [10] from India also revealed a high prevalence of luminal A subtype (63%) and a low prevalence of triple negative-like subtype (8%) in breast cancers, although an age-specific variation was not described. 

A comparison of various studies on molecular subtyping in young breast carcinoma has been described in Table 6.

**Table 6:** Prevalance of Luminal A subtype in the young carcinoma breast in various studies

Author, Year, Country	Age criteria for classifying as young carcinoma breast	Prevalence of Luminal A
Collins et al, 2012, USA [11]	≤40 years	33.0%
Goksu SS et al, 2014, Turkey [12]	≤35years	31.0%
Lin et al, 2009, Taiwan [8]	≤50 years	67.0%
Our study, 2018, India	≤40 years	59.2%

In contrast to the women in the United States (US), younger women with breast carcinoma in Asia did not have worse outcomes compared to older women. This occurred, in spite of more advanced disease at diagnosis and higher grade tumors, suggesting an ethnic and environmental variation, a possible etiology for outcome discrepancies [13].

Asian women have been described to have a relatively better outcome in terms of survival and prognosis as compared to African, Latin, and non-Hispanic native American women [14-15]. The possible reason could be a high prevalence of the lethal triple-negative phenotype (ER−, PR−, HER2−) in young African-American women [16]. These observations suggest that ethnic differences in breast carcinogenesis exist among Asian and Occidental populations [8]. Therefore, genetic factors or their interaction with other environmental factors may contribute to the observed variation of young breast carcinogenesis in India and other parts of Asia.

Along with molecular subtyping, we correlated other prognostic factors, like lymph node metastasis and lymphovascular invasion, to strengthen our findings and for a better description of the pathogenesis. Lymph node metastases were more common in the younger group compared to the older age group, which reemphasizes the aggressive nature of young breast carcinoma and is consistent with a study done by Anders et al. [17].

HER2 molecular subtype was associated with the highest percentage of lymphatic metastases, which is comparable with a study done by Tokatli et al. [18]. Higher lymphovascular invasion in younger age group, suggestive of aggressive disease, was also in agreement with a study done by Lee et al. [19].

The strength of our study is a comprehensive analysis of the molecular pathology of young breast carcinogenesis in Indian women, highlighting its distinctive behaviour. However, the study is limited by small sample size and lack of inclusion of the Ki-67 marker for molecular subtyping due to financial constraints.

## Conclusions

Molecular classification assessment with the aid of IHC is highly informative and should be adopted as a part of routine diagnosis in the management of breast carcinoma patients. Hormonal (ER, PR) and HER2 status elucidation not only helps in assessing the prognosis but is also useful for predictive analysis. This study throws light on the correlation of molecular subtyping and age in Indian women, an area largely unexplored in clinical research. As opposed to the prevalent Western literature showing breast carcinoma in the younger group as a more aggressive disease, the results of our study are in accordance with the Asian literature, showing no significant difference in molecular subtyping of breast carcinoma in the two age groups.
